# Effect of 3D-printed co-culture design of mesenchymal stem cells and human umbilical vein endothelial cells on tubular formation

**DOI:** 10.1038/s41598-026-55959-y

**Published:** 2026-06-08

**Authors:** Åshild Johansen, Jannika T. Korkeamäki, Shuntaro Yamada, Ahmad Rashad, Susanna Miettinen, Kamal Mustafa

**Affiliations:** 1 Department of Clinical Dentistry, Center of Translational Oral Research (TOR), Tissue Engineering Group, University of Bergen, Årstadveien 19, Bergen, 5009 Norway; 2 Faculty of Dentistry, Oral and Craniofacial Sciences, Centre for Craniofacial and Regenerative Biology, King’s College London, London, UK; 3 UNAM-Institute of Materials Science and Nanotechnology, The National Nanotechnology Research Center, Bilkent University, Ankara, 06800 Turkey; 4 Faculty of Medicine and Health Technology, Adult Stem Cell Group, Tampere University, Tampere, Finland; 5 Tays Research Services, Wellbeing Services County of Pirkanmaa, Tampere University Hospital, Tampere, Finland

**Keywords:** HUVEC, BMSC, Co-culture, Vascularization, Pericyte, Tissue engineering, Bioprinting, Biological techniques, Biotechnology, Cell biology, Materials science, Stem cells

## Abstract

Functional vascularization remains a major challenge in engineered tissues. Multi-material 3D bioprinting enables precise spatial patterning of different cell types, offering new opportunities to design engineered microenvironments that support vascular self-assembly. Here, we compared three bioprinted co-culture configurations of human umbilical vein endothelial cells (HUVEC) and bone marrow–derived mesenchymal stem cells (BMSC) using a fibrin-gelatin bioink: (i) co-printed within the same filament, (ii) printed in adjacent but distinct filaments, and (iii) cultured in paracrine mode without direct contact. Viability, metabolic activity, and endothelial network formation were evaluated over 14 days. While all designs maintained high viability and metabolic activity, only configurations incorporating BMSC supported extensive and stable CD31-positive endothelial networks. Quantitative 3D surface analysis revealed significantly greater tubular surface area in both co-printed and adjacent filament constructs versus paracrine-only conditions. Notably, HUVEC network formation in adjacent filament constructs was comparable to that achieved by co-printing, indicating that initial physical co-localization within the same filament is not required, provided cells are within migratory distance. These findings establish a practical design principle for bioprinted vascularized constructs: physical proximity, rather than filament co-localization, is sufficient to promote endothelial self-organization, thereby expanding design flexibility for multi-material bioprinting strategies.

**Supplementary Information:**

The online version contains supplementary material available at 10.1038/s41598-026-55959-y.

## Introduction

 The discrepancy between the demand for tissue and organ transplants and their availability continues to increase. This gap underscores the urgent need for laboratory-grown, tissue-engineered constructs and organs^1^. In addition to restoring the function of the target tissue, adequate blood supply is crucial for the survival of a transplant, as blood vessels are essential for nearly all tissues, serving as exchange sites for nutrients and oxygen, as well as for the removal of waste products and carbon dioxide^2^. In most tissues, the distance from a cell to the nearest vasculature should not exceed approximately 200 μm, which represents the diffusion limit of oxygen^3,4^. A greater distance, or the absence of sufficient vasculature, can impede cell proliferation, differentiation, and overall tissue viability, which ultimately can lead to transplant failure^2^. These considerations highlight the importance of incorporating vascularization strategies when engineering larger tissue constructs.

Additive manufacturing has emerged as a prominent fabrication technique of tissue-engineered implants, offering superior precision in the fabrication of patient-specific, tailor-made scaffolds^5,6^. In particular, 3D bioprinting represents a significant advancement, enabling precise layer-by-layer deposition of bioinks and allowing for controlled, homogeneous cell distribution throughout the construct. Moreover, this technology supports the simultaneous printing of multiple bioinks, each potentially containing distinct cell types, facilitating the fabrication of complex, multi-material constructs, with the potential to mimic different tissues^6,7^. These advances also open new possibilities for co-culture systems, in which distinct cell types can be incorporated within a single bioink, within the same object.

However, several reported 3D bioprinting strategies lean heavily on designed hollow structures, the top-down approach, instead of leaning on to the natural spontaneous neovascularization potential of embedded vascular endothelial cells (EC), namely the bottom-up approach^8–11^. The top-down approach creates limitations, increasing the diameter according to the fabrication method accuracy, which, in the case of extrusion bioprinting, is often limited by the nozzle diameter typically being in the 100 μm range, thus greatly exceeding the capillary diameter up to 10 μm^8,12,13^. While synthetic tissue engineered vascular grafts have been successful in large diameter applications, representing larger arteries (100−10,000 μm) and veins (> 100−10,000 μm), majority is reported to fail with smaller diameters, representing capillaries (4–10 μm), and arterioles and venules (10–100 μm), due to the thrombogenic tendency of the absence of the endothelial layer^14,15^. Therefore, the printing of hollow structures in the capillary range remains a challenge, underscoring the importance of the bottom-up approach, with self-assembly of EC in situ, allowing formation of microvascularization^4,16^. In this context, the self-assembly of HUVEC leads to the formation of an endothelial lining from an early stage, which is inherently anti-thrombogenic and could potentially mitigate clot formation^17^.

Blood vessels are lined by a continuous monolayer of EC that forms a semipermeable barrier for molecular exchange, while the surrounding extracellular matrix (ECM) provides structural support and hosts perivascular cells such as pericytes (PC)^2,18^. In vitro vascular tissue engineering leverages the intrinsic ability of endothelial cells to self-assemble into vessel-like structures^19^. However, there is substantial evidence that the inclusion of pericyte-like supporting cells enhances the robustness and maturation of these vascular networks^20^. Direct co-culture of EC with stromal cells has proven to be an effective approach for promoting the formation of more mature and stable vasculature in vitro, with stromal cells acting as pericyte analogues that contribute to microvascular structure and function^20–22^. However, it is also reported that the paracrine effect of a co-culture could be adequate to support the formation and stability of tubular networks^23^.

In this study, three 3D bioprinted co-culture systems were explored to evaluate how different co-culture designs influence the self-assembly of tubular networks. The supporting role of bone marrow-derived mesenchymal stromal cells (BMSC) within a 3D bioprinted construct and their contribution to neovascularization were also assessed. Three co-culture designs were investigated: (I) *Direct [mixed] (DM)*, where human umbilical vein endothelial cells (HUVEC) and BMSC were bioprinted together within the same filament; (II) *Direct [separated] (DS)*, where HUVEC and BMSC were bioprinted in adjacent but compositionally distinct filaments, enabling assessment of cell migration; and (III) *Indirect [paracrine] (I)*, where HUVEC were embedded in the bioink while BMSC were seeded in a hanging cell culture insert to provide paracrine signaling without direct physical contact. These configurations were designed to investigate how spatial organization of endothelial and stromal cells influences cell–cell interactions and vascularization outcomes. As cells within biofabricated constructs may be positioned either in direct contact or spatially separated, resulting primarily in paracrine signaling, systematically comparing these co-culture approaches is essential for optimizing construct design to support vascular network formation.

The same bioink composition was used for all groups, and the same HUVEC: BMSC cell ratio was maintained. To facilitate the co-culture investigation, a fibrin-gelatin bioink was developed to support angiogenesis and is hereafter referred to as “vascu-ink”. Fibrin, a natural polymer that promotes angiogenesis and participates in wound-healing processes, was selected to serve as a temporary extracellular scaffold^1,24,25^. Gelatin, a collagen derivative, was included for its thermosensitivity, which improves printability and structural stability^1,24,26^. The gelatin in the bioink was not crosslinked and therefore primarily acted as a sacrificial component, as it returns to a liquid state under cell culture conditions and gradually diffuses out of the construct^27^. While some gelatin may remain transiently entrapped within the matrix, fibrin constitutes the primary structural component under culture conditions. Given this enzymatically crosslinked fibrin-based system, an extrusion-based 3D printing approach was selected, as it enables deposition of the bioink prior to thrombin-mediated conversion of fibrinogen to fibrin and is well-suited for this material composition and experimental design, including the use of multiple bioinks within a single construct.

## Materials and methods

### Material preparation

Vascu-ink was prepared by dissolving fibrinogen (F8630; Sigma Aldrich, USA) in Dulbecco’s Phosphate Buffered Saline (DPBS) (59300 C; Merck, US) to reach a final concentration of 2% (w/v). A 32% (w/v) gelatin stock solution (G2500; Sigma Aldrich, USA) was prepared separately by heating to 75 °C while stirring at 50 rpm. The gelatin stock was then added to the fibrinogen solution to obtain a final concentration of 4% (w/v). The ink mixture was maintained at 37 °C under gentle stirring at 50 rpm. Both fibrinogen and gelatin powders were sterilized by ultraviolet (UV) exposure for 1 h prior to ink preparation.

### Rheological characterization

The rheological properties of the biomaterial inks were measured using a Discovery HR-2 rotational rheometer (TA instruments, New Castle, DE, USA). Biomaterial ink samples were placed between parallel plates with a diameter of 20 mm, 1500 μm gap. They were measured for their viscosity under increasing shear rate (0.1–300 s^− 1^) at a constant temperature (22 °C), and under decreasing temperature (37–20 °C) at a constant shear rate (1/s). Before measurement, the samples were allowed to reach equilibrium for 5 min to minimize the effects of sample handling. All measurements were performed using a solvent trap to avoid water evaporation during the test. Biomaterial ink was measured in *n* = 4 independent replicates to assess reproducibility and identify potential outliers. The data shown in the figures are representative of these replicates, which exhibited consistent rheological behavior. Viscosity at printing temperature was assessed using n_datapoints_= 20 (22 °C ± 0.2 °C).

### Cell culture

With informed consent, HUVEC were isolated from umbilical cords obtained after planned cesarean sections, as previously described, under ethical approval from the Ethics Committee of the Pirkanmaa Hospital District, Tampere, Finland (R13019)^28^. All experiments were performed in accordance with relevant guidelines and regulations. HUVEC were cultured in EGM^®^-2 Endothelial Cell Growth Medium-2 BulletKit (EGM-2: CC-3162; Lonza, Switzerland). The fetal bovine serum (2%) provided in the kit was replaced with 2% human serum (HS) (S419; BioWest, France), and the medium was supplemented with 1% penicillin and streptomycin (PS) (SV30010; HyClone, US).

BMSC were obtained from anonymous donors undergoing orthopedic surgery after written informed consent. The cells were harvested and isolated as previously described, under ethical approval from the Ethics Committee of the Expert Responsibility area of Tampere University Hospital, Tampere, Finland (R15174)^28^, and the experiments were performed in accordance with relevant guidelines and regulations. BMSC were cultured in α-minimum essential medium (α-MEM: 22 571; Gibco, USA) supplemented with 5% HS, 1% PS, and 5 ng/ml human fibroblast growth factor (130-093-838: Miltenyi Biotec, Germany).

Cells were maintained at 37 °C in a humidified atmosphere containing 5% CO_2_ and used at passage 4–5. In this study, 1 BMSC donor and 1 HUVEC donor were used.

The BMSC were confirmed to exhibit multipotent characteristics by osteogenic and adipogenic differentiation, as previously reported^29^. Both BMSC and HUVEC were verified as cells of mesenchymal origin and endothelial cell types, respectively, by surface marker expression, as previously described and reported. Briefly, BMSC were characterized as MSC by positive expression of CD73, CD90, and CD105, and low or negative expression of CD14, CD19, and CD45. CD34 and HLA-DR was present at variable levels^29^. Endothelial phenotype of HUVEC were confirmed through high expression of CD31, CD73, CD105, and CD146, and moderate expression of CD34, CD36, CD144, CD202B, and VEGFR3, and low expression of CD90 and VEGFR2^28^.

### Bioprinting

**Fig. 1:**
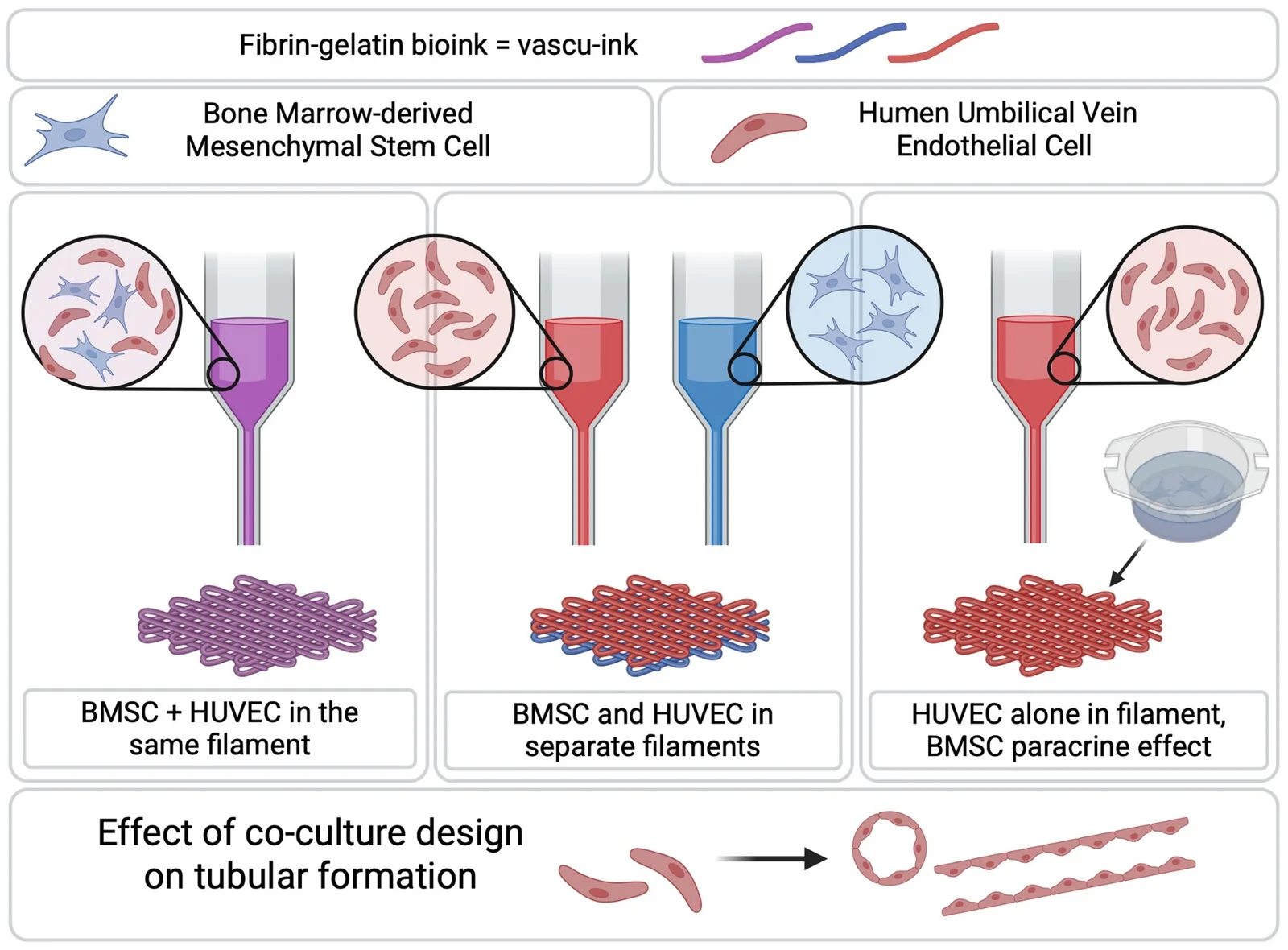
Schematic illustration of the study design showing the three different co-culture configurations of HUVEC and BMSC: (i) co-printed within the same filament (i.e., *Direct [mixed]*), (ii) printed in adjacent but distinct filaments (i.e., *Direct [separated]*), and (iii) cultured in a paracrine configuration without direct cell-cell contact (i.e., *Indirect*).

The vascu-ink was preheated to 37 °C prior to cell addition. HUVEC and BMSC were trypsinized and added to the pre-filtered vascu-ink at a volume of 50 µl/mL, corresponding to a total cell density of 10 million cells/mL. A HUVEC: BMSC ratio of 5:1 was used, based on previous work^21,30^. This yielded 8.33 million HUVEC and 1.67 million BMSC per mL in the *Direct [mixed]* (DM) group. For the *Direct [separated]* (DS) group, two bioinks were prepared, containing 10 million HUVEC/mL and 2 million BMSC/mL, respectively, resulting in a lower initial cell number in this group. For the *Indirect [paracrine]* (I) group, HUVEC were mixed into the bioink at a concentration of 10 million cells/mL, while BMSC were seeded in hanging cell culture inserts (0.3 cm²; Millicell^®^ 24-well inserts, PTHT24H48, Merck Millipore, USA) at a density of 80,000 cells per insert, corresponding to approximately 267,000 cells/cm², to maintain an overall 5:1 cell ratio within each sample (Fig. 1).

The cell-laden vascu-ink was gently mixed and transferred into sterile plastic printing cartridges fitted with a Ø_ID_ = 0.25 mm nozzle (7018345; Nordson EFD, USA). The cartridges were cooled at 4 °C for 18 min before printing. Printing was performed using a 3D-Bioplotter (EnvisionTEC, Germany) and the temperature set to 21–23 °C, allowing 30 min for temperature equilibration. A 3D CAD cylinder model (Ø = 10 mm) was generated using Magics software (EnvisionTEC, Germany) and sliced into 80% of the inner diameter of the extrusion needle. Constructs consisting of four layers (0.75 mm filament distance, 0/90° angle) were printed with a pressure of 1.0–2.3 bars and a speed of 12.5–17.7 mm/s. Samples were printed directly into sterile 24-well plates placed on a 4 °C cooled platform. Following printing, constructs were crosslinked with 2.5 U/ml Thrombin (T7513; Sigma-Aldrich, USA) in 25 mM CaCl_2_ solution (Calcium chloride dihydrate: C5080, Sigma-Aldrich, USA) for 10 min, followed by two 5 min washes with DPBS. The samples were cultured in EGM-2 medium supplemented with 2% HS, 1% P/S and 100 µg/ml Normocin (ant-nr-1; InvivoGen, USA). A total of 1.5 mL medium was added to each well, and the medium was changed three times per week. The experiment was conducted twice.

### Viability and metabolic activity

Cell viability after printing was assessed using the Live/Dead staining kit (L3224; Invitrogen, USA) according to the manufacturer’s instructions. Samples were incubated in the staining solution for 45 min at room temperature (RT), washed with DPBS, and imaged using a fluorescent microscope (Nikon Eclipse Ti; Nikon, Japan).

Metabolic activity, serving as an indirect measure of cell proliferation, was evaluated using the Cell Counting Kit-8 (CCK-8CK: 04–11; Dojindo Laboratories, Japan). Samples were washed with DPBS and then incubated in a 10:1 mixture of cell culture medium and undiluted CCK-8 reagent for 2 h at 37 °C in a humidified atmosphere containing 5% CO_2_. After incubation, the CCK-8 solution was transferred to a 96-well plate and measured in triplicate. Absorbance was recorded at 450 nm using a microplate reader (VarioskanTM LUX; VLBL00D0: Thermo Scientific, USA). The same samples (*n* = 6) were used across all time points. Following absorbance measurements, the samples were washed with DPBS and replenished with fresh culture medium. Viability and metabolic activity were assessed on day 1, day 3, day 7, and day 14 after printing.

### Immunohistochemical staining

Prior to immunostaining, samples were fixed in a 4:1 ratio of paraformaldehyde (PFA) 4% and 100 mM CaCL_2_ for 60 min at RT, followed by two 10 min washes in DPBS. Samples were stored in DPBS containing 10 mM CaCl_2_ until further processing. Samples were permeabilized with 0.3% Triton-X in DPBS for 10 min at RT and subsequently blocked overnight at 4 °C on a shaker in blocking solution containing 10% normal goat serum (NGS) (G9023, Sigma-Aldrich, USA), 0.1% Triton-X, 1% bovine serum albumin (BSA) (A9647; Sigma-Aldrich, USA) and 10 mM CaCl_2_ in DPBS. Following blocking, samples were washed for 15 min in 1% NGS, 0.1% Triton-X, 1% BSA and 10 mM CaCl_2_ in DPBS. Primary antibodies were diluted in staining solution containing 1% NGS, 0.1% Triton-X, 1% BSA, and 10 mM CaCl_2_ in PBS, and samples were incubated for 2 days at 4 °C on a shaker. The following primary antibodies were used: CD31 mouse monoclonal (1:200, M082301-2; Agilent Dako, USA), CD31 mouse monoclonal CoraLiteⓇ 594 (1:250, CL594-66065; ProteinTech, USA), von Willenbrand factor (vWF) rabbit polyclonal (1:150, ab9378; Abcam, UK), α-smooth muscle actin (α-SMA) mouse monoclonal Alexa Fluor™ 488 (1:250, 53-9760-82; Invitrogen, USA), and Collagen IV (Col-IV) mouse monoclonal (1:200, C1926; Sigma-Aldrich, USA). After primary incubation, samples were thoroughly washed four times in DPBS containing 0.1% Triton-X, 1% BSA, and 10 mM CaCl_2_. Counterstaining with DAPI (1:2500; D9542; Sigma-Aldrich, USA) and Phalloidin-Atto 565 (1:250, 94072; Sigma-Aldrich, USA), together with incubation with secondary antibodies, was performed overnight at 4 °C on a shaker in staining solution containing 1% BSA and 10 mM CaCL_2_ in DPBS. The secondary antibodies used were goat anti-mouse Alexa Fluor™ 488 (1:400, 10256302; Invitrogen, USA) and goat anti-rabbit AlexaFluor™ 647 (1:400, 10123672; Invitrogen, USA). Finally, samples were thoroughly washed four times with 10 mM CaCl_2_ in DPBS on a shaker at 4 °C. Imaging was performed inside the sample, and image acquisition was performed using a confocal laser scanning microscope (TCS SP8 STED 3X confocal, Leica, Germany). For samples stained for CD31, adaptive deconvolution was applied using the Lightning software module.

### Chorioallantoic membrane assay

Fertilized eggs (*Gallus gallus domesticus*) were obtained from a local farmer and incubated in an automated egg incubator (Willab AB; Sweden). At embryonic day 3 (E3), the eggs were carefully cracked, and those with intact yolk and chorioallantoic membrane (CAM) were kept in sterile plastic containers containing 2 ml DPBS supplemented with 1% P/S. The eggs were incubated at 37 °C in a humidified environment. Blank and cell-laden bioprinted vascu-ink (DM) samples were pre-incubated for 5 days and subsequently placed onto the CAM surface at embryonic day 7 (E7). The CAM and samples were fixed at embryonic day 12 (E12) with 4% PFA for further analysis. For histologic analysis, dissected samples were dehydrated through a graded ethanol series (70%, 80%, 95%, and 100%) for 1 h per step, followed by clearing in xylene for 2 h. Samples were infiltrated with molten paraffin wax at 60 °C in two changes, and embedded in paraffin blocks. Using a microtome (RM2155, Leica, Germany), the blocks were trimmed and sectioned at 4–5 μm thickness. Sections were floated on a 40 °C water bath, mounted onto glass slides, and dried overnight at 50 °C. Unstained sections were cleared in xylene followed by rehydration through a graded ethanol series. The slides were then stained with hematoxylin and eosin (H&E) and imaged using a light microscope (Eclipse 80i; Nikon, Japan). Additionally, immunofluorescent staining was performed targeting human CD31 and αSMA as mentioned above following heat induced antigen retrieval using sodium citrate, pH 6.0 for 20 min at 95 ℃.

### Statistical analysis and data illustration

Image processing and visualization of immunostained and Live/Dead stained samples were performed using FIJI/Image J (Oxford instruments, UK). Statistical analyses were conducted in Prism 10 (Dotmatics, USA), and data are presented as mean ± standard deviation (SD). For the metabolic activity data, a two-way analysis of variance (two-way ANOVA) followed by Tukey’s multiple comparisons test was performed. A p-value < 0.05 was considered statistically significant.

For the CD31 immunostained samples, 3D image surface rendering and quantification were performed using IMARIS (Oxford Instruments, UK). All images were cropped to 259 × 259 × 96 μm and processed with a Gaussian filter and layer normalization. Surface rendering parameters, including surface details, threshold, and filtering, were applied identically across all samples. Quantitative surface area data were extracted from each 3D image. Quantitative data of the tubular structure surface area were analyzed using Prism 10, where violin plots were generated, and a Kruskal-Wallis test followed by Dunn’s multiple comparisons test was performed. Data illustration of histograms was conducted in R, using the tidyverse, readxl, and ggplot2. From one or two biological replicates, four to eight regions of interest (ROIs) were analyzed per condition, (*n* = 4–8).

## Results

### Rheology and printing

**Fig. 2:**
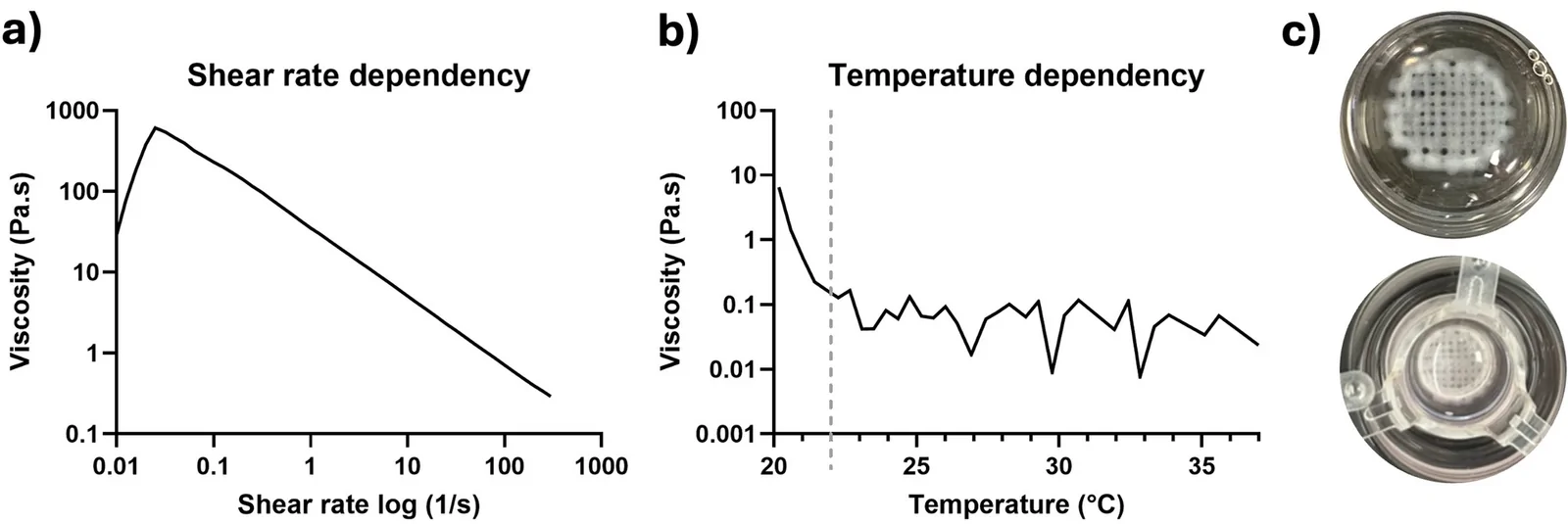
Rheology of vascu-ink showing (**a**) Shear rate dependency and (**b**) Temperature dependency, with a stipulated grey line marking the desired printing temperature. (**c**) Printed samples in their wells after cross-linking, and the lower well with the hanging cell-culture insert.

The results of the biomaterial ink rheological characterization are presented in Fig. 2 and S1. Regarding shear rate dependency (Fig. 2a), the viscosity first increased and then rapidly decreased when the shear rate increased, demonstrating both shear-thickening behavior at a low shear rate and shear-thinning behavior at a higher shear rate. In terms of temperature dependence, first, a gradual increase in viscosity was observed as temperature decreases (Fig. 2b). However, the viscosity increased rapidly as the biomaterial ink cooled below 22 °C. The viscosity of the biomaterial ink at printing temperature was determined to be 0.173 ± 0.055 Pa.s. Four-layer cylinders were printed with a 0.75 mm filament spacing, with each successive layer oriented 90° to the previous one, which preserved pore integrity. The printed structures remained stable in culture (Fig. 2c). The vascu-ink was evaluated with different needle sizes, which revealed the potential to be extruded with a needle thickness ranging from 0.15 mm to 0.5 mm (Fig. S2a).

### Bioprinted co-cultures demonstrated high cell viability and increasing metabolic activity

**Fig. 3:**
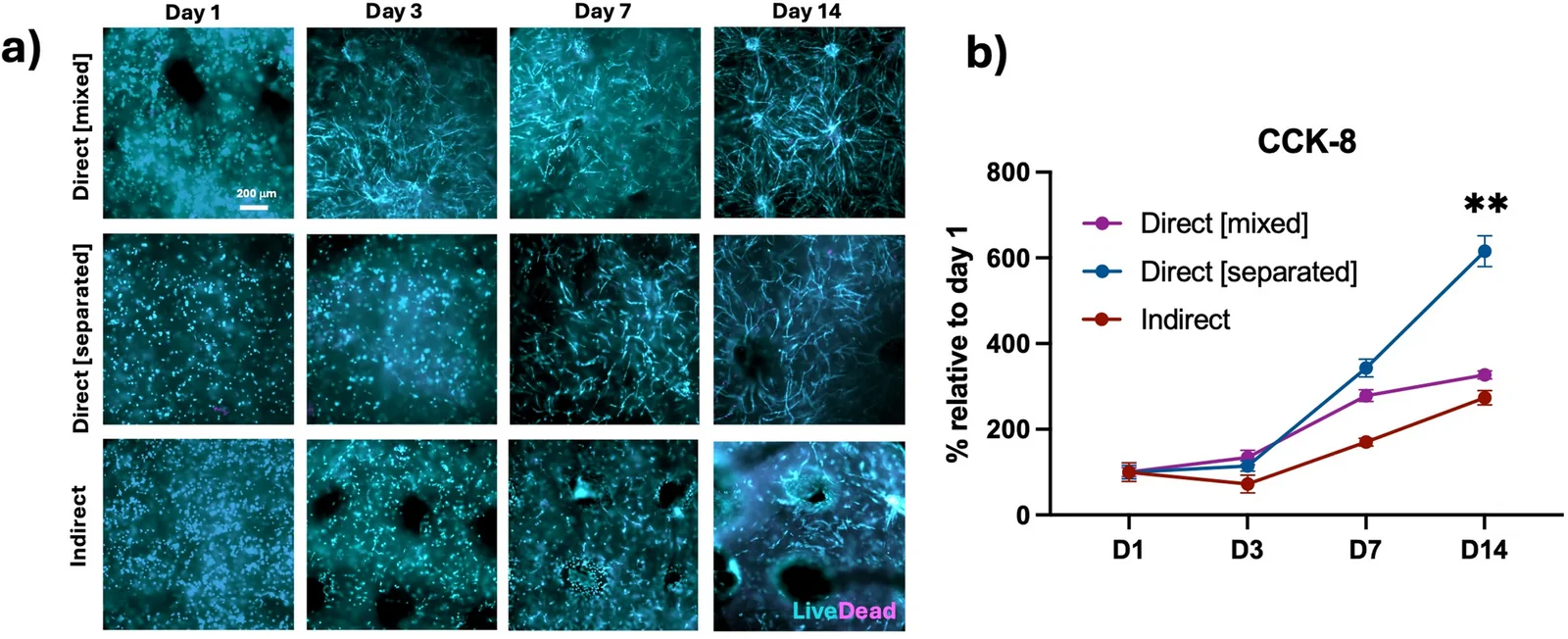
(**a**) Live/dead staining of bioprinted vascu-ink with HUVEC and BMSC in *physical contact* and *separate filament* group, and HUVEC in *paracrine effect* group. (**b**, **c**) Metabolic activity of BMSC and HUVEC measured by CCK-8, as shown by absorbance relative to day 1 in percentage. *n* = 6 for all groups and time points, data presented as mean with error bars representing SD. **p < 0.01 separate vs. paracrine (two-way ANOVA).

High cell viability was observed on day 1 after bioprinting and maintained throughout the 14-day culture period, with only a minimal number of dead cells detected (Fig. 3a). On the contrary, samples with only HUVEC and without BMSC, showed adequate viability during the initial days of culture, but with increased culture time, more dead cells were qualitatively assessed, and a lower cell density was observed by the end of two-week culture period (Fig. S4). HUVEC network formation, along with cell elongation and spreading, was evident over time, starting with round cells at day 1 and progressively more organized network formation at later time points, particularly in groups containing embedded BMSC. Metabolic activity increased over time across all groups. However, a greater increase was observed in the *Direct* groups where BMSC were physically present (Figs. 3b, S3).

### The presence of BMSC enhanced the stability of tubular structures

**Fig. 4:**
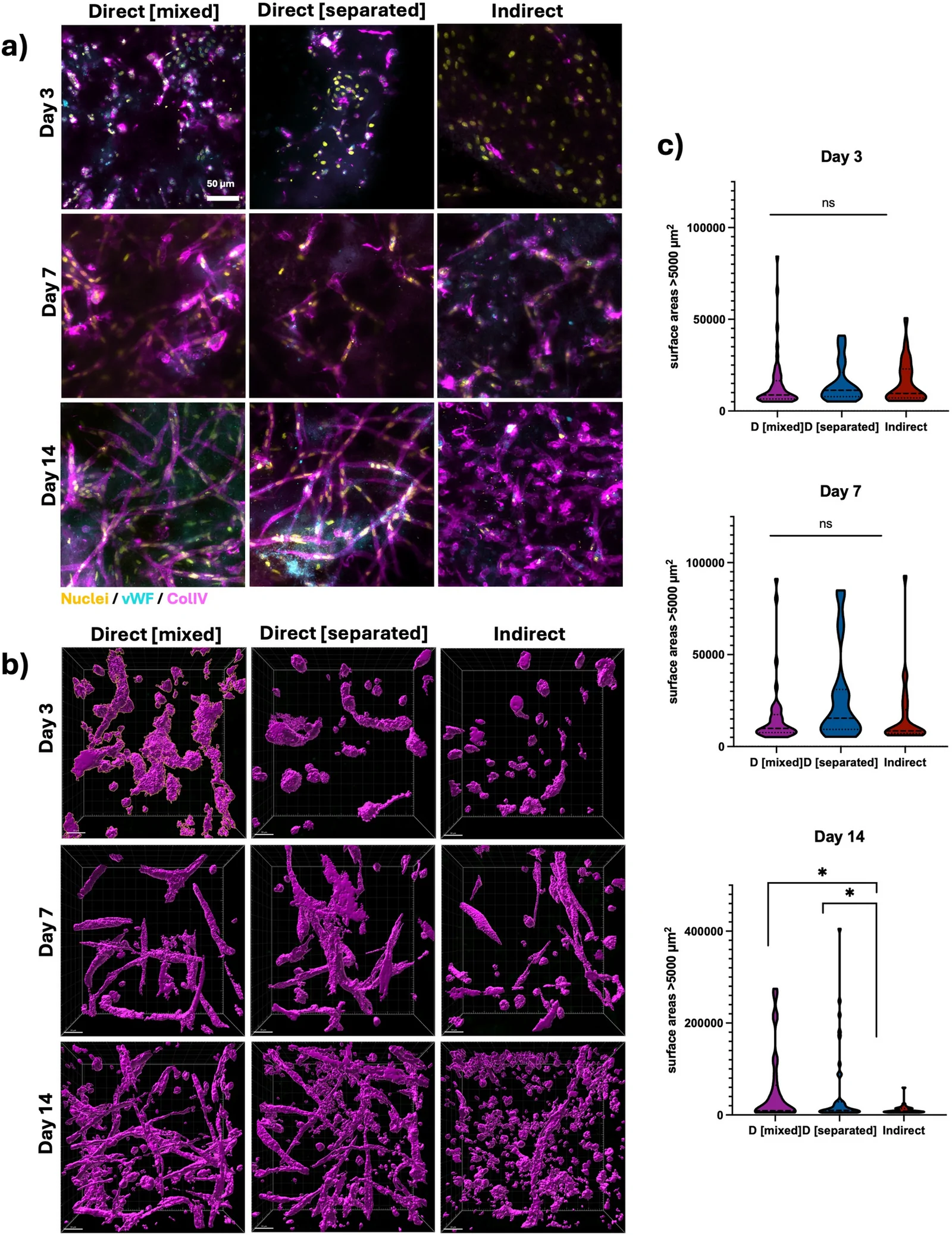
(**a**) Immunostaining of 3D-bioprinted samples, showing the formation of tubular structures by staining for Collagen IV, von Willebrand factor, and DAPI, after 3, 7, and 14 days. The *Direct [mixed]* and *Direct [separated]* bioprinted samples contain HUVEC and BMSC, and the *Indirect* group sample contains HUVEC. (**b**) 3D-images immunostained for CD31 and surface rendered in IMARIS. Scalebar = 30 μm. (**c**) Violin plots showing the distribution of surface areas larger than 5000 µm^2^.

The immunohistochemical staining revealed distinct differences among the three groups after 14 days of culture. Immunostaining for Col-IV, a major structural component of the basement membrane that supports capillaries and vasculature, shows slight aggregation of HUVEC three days after bioprinting, but by day 7, clear organization is observed in all groups. However, by day 14, tubular network structures appeared longer and more distinct only in the groups containing the encapsulated BMSC (Fig. 4a). Moreover, the 3D rendering and surface reconstruction executed through immunohistochemical staining of CD31, an endothelial cell-cell junction protein, showed larger networks in the *Direct* groups (Fig. 4b). The quantitative analysis of surface area distributions was in line with the qualitative assessment, demonstrating only minor differences for days 3 and 7 (Figs. 4c, S4), and a significant difference by day 14, as several large surface areas exceeding 250,000 µm^2^ were observed both in the *Direct* groups, in comparison to the largest surface area in the *Indirect* group, being around 60,000 µm^2^.

### Cells migrate between bioprinted filaments

**Fig. 5:**
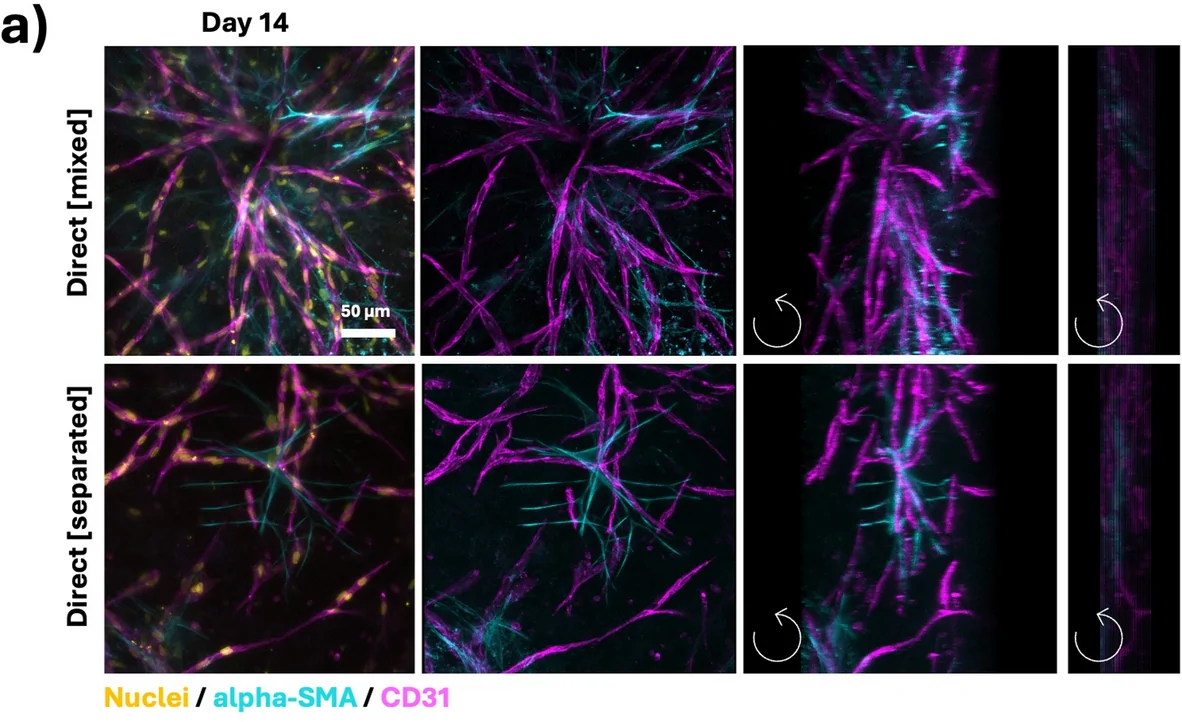
(**a**) Immunostaining of alpha-SMA and CD31 shows the position of BMSC at 2D, but also in 3D, by rotating the 3D-image ∼45 and 90 degrees.

To investigate the positioning and potential migration of cells in the two BMSC embedded models, the *Direct* groups were immunohistochemically stained for CD31 and α-SMA, a marker for vascular pericytes (Fig. 5). In the captured 3D images, the results demonstrated α-SMA labeling among the CD31 labeling, representing BMSC and HUVEC respectively, in both groups. This indicates cell migration in the *DS* group.

### Proof-of-concept histological analysis demonstrates lumen formation in tubular structures

**Fig. 6:**
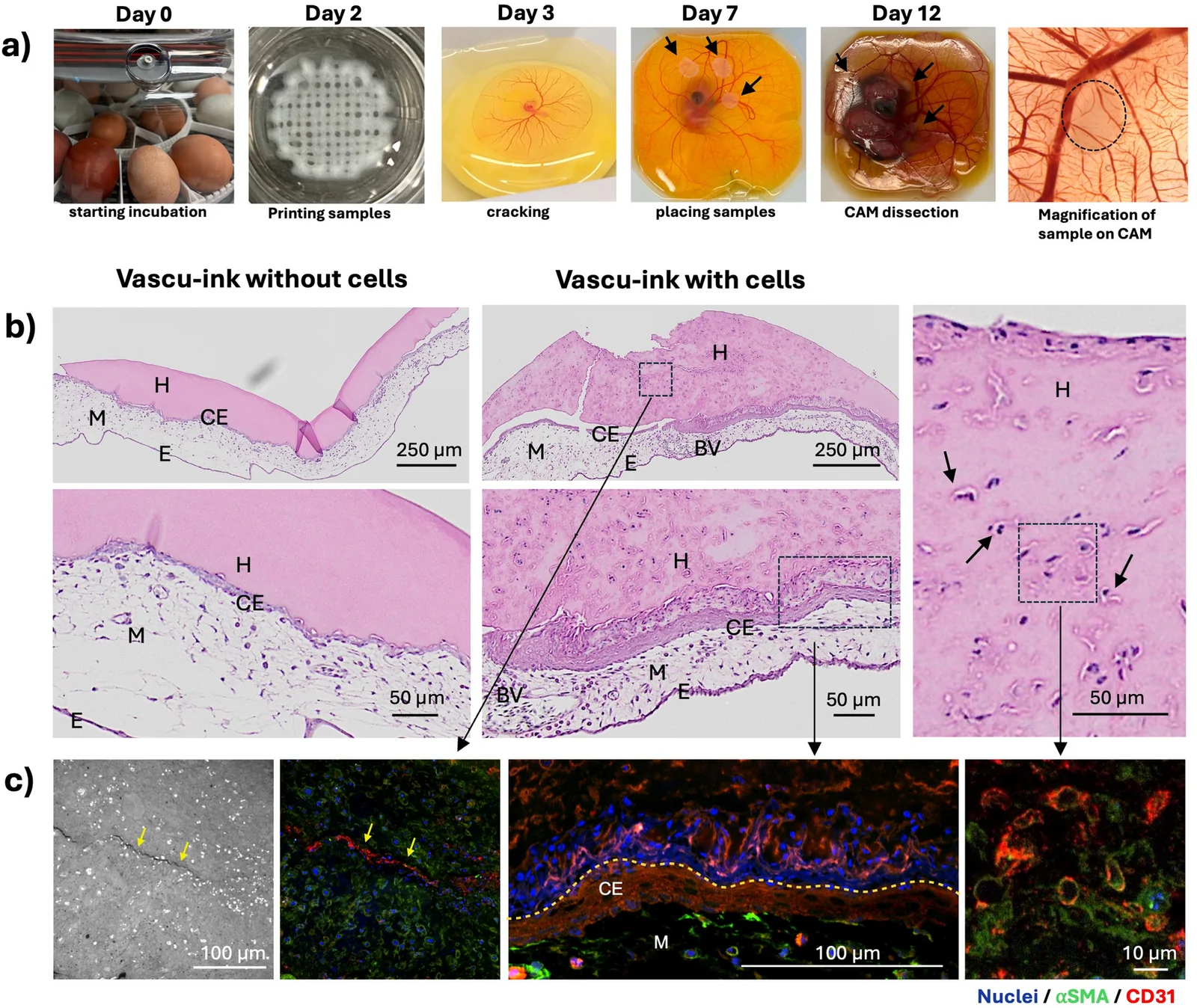
(**a**) Overview of the events of testing bioprinted structures on the chorioallantoic membrane (CAM). (**b**) Hematoxylin and eosin staining, and (**c**) immunohistochemical staining for alpha smooth muscle actin and CD31 of dissected bioprinted samples on the CAM. To the left, a crevice-like macro vessel; in the middle, sprouting endothelial cells towards the CAM; to the right, micro-lumens. H = hydrogel, CE = chorionic ectoderm, E = allantoic endoderm, M = mesenchyme, BV = blood vessel.

Histological evaluation showed that the printed constructs were well integrated into the CAM, confirming the biocompatible nature of vascu-ink, both with and without incorporated cells (Fig. 6a, b). The absence of inflammatory responses in the surrounding CAM tissue further supported the biocompatibility of vascu-ink. Examination of the cellular vascu-ink samples revealed the presence of lumens (≤ 10 μm) within the tubular structures formed by HUVEC, demonstrating the capacity of the system to support vascular-like organization in this proof-of-concept setting (Fig. 6b, c). Immunohistochemical staining further revealed a larger, crevice-like CD31-positive structure resembling a macro vessel. Interestingly, the HUVEC in the vascu-ink sample adjacent to the CAM appeared to exhibit sprouting behavior directed toward the CAM tissue.

## Discussion

To advance the neovascularization of engineered tissue constructs, it is essential to develop a solution that mimics or supports the formation of microvasculature. Therefore, in this study, we investigated the potential of three co-culture designs for their ability to self-assemble tubular networks while embedded in a 3D bioprinted gelatin-fibrin construct. The co-culture configurations were assessed in terms of bioink potential as well as the biological effects of BMSC proximity to HUVEC. Notably, HUVEC network formation in adjacent filament constructs was comparable to that observed in co-printed constructs, demonstrating that initial direct physical contact within the same filament is not essential, provided that the cells remain within migratory distance.

High viability and increased metabolic activity, indicative of proliferation, were observed in all three groups. However, the *DS* group had the steepest increase in metabolic activity, which could be explained by the lower initial cell number in the samples, since high initial seed densities could influence the nutrient availability^31^. In this setting, the HUVEC: BMSC cell ratio (5:1) was maintained across all groups; however, the total cell number per sample differed between groups. Total cell number is known to influence paracrine signaling, as increased cell density can lead to cumulative secretion of bioactive factors^32^. To maintain the defined cell ratio, a relatively high seeding density was used in the cell culture inserts in the *Indirect [paracrine]* group. While BMSC viability within the inserts was not directly assessed, high cell density may influence cellular behavior. Additionally, high-density conditions may promote localized hypoxia within the inserts, which has been associated with increased expression of pro-angiogenic factors such as VEGF^33^. The placement of BMSC did not affect the viability or proliferation of embedded HUVEC; however, bioprinted samples with only HUVEC revealed a decreased viability and fewer cells visible in the ink with culture time^34,35^.

Where high viability and proliferation were observed across all groups, the HUVEC self-organization capability was more clearly affected by the availability of BMSC. There are three main mechanisms of communication between EC and MSC. The first involves direct interaction between adjacent cells through membrane-bound molecules, which requires physical contact. The other two mechanisms occur without direct contact, through signaling via diffusible soluble factors that bind to specific receptors on the target cell, and through the release and uptake of extracellular vesicles (EVs) carrying bioactive molecules^36,37^. A significant difference was observed after 2 weeks of culture: the *Direct* groups showed long tubular structures, whereas the *Indirect [paracrine]* group showed more fragmented cell clusters. In comparison, no distinct network could be identified in the HUVEC control. The quantified difference between the largest structure in *Direct [separated]* and the largest structures in *Indirect [paracrine]* was 6.8-fold. This is in line with previous findings that the physical co-culture of supporting cells will yield more mature and robust vasculature^1,20^. In the *Indirect* group, the available communication pathways between HUVEC and BMSC were limited to soluble factors and EVs. This restricted communication, combined with the absence of physical support from BMSC, may explain the reduced stability and maturation of the HUVEC networks observed after 14 days. However, the *Indirect* group showed greater HUVEC assembly and more cells in the ink after 14 days of culture than HUVEC alone. This indicated that the paracrine effect from BMSC has an impact to some degree, and some report stable and mature vessels with only the paracrine effect from pericytic cells, however, our results show that it is not comparable to the impact of pericytes being physically present^23,36^. However, HUVEC alone was not enough to form mature, stable vessel structures, and little HUVEC organization was seen, as described by previous findings^20,38,39^.

As the presence of the embedded BMSC was determined to be crucial for the longevity and stability of the tubular networks, the proximity of the BMSC in relation to the HUVEC was investigated. In the *DS* group, HUVEC and BMSC were located in distinct but closely adjacent filaments to investigate the migration potential of cells within vascu-ink. After 14 days of culture, immunostaining for α-SMA showed positive staining in the same Z-stack as CD31-labelled HUVEC in both *DM* and *DS* groups. Cell migration is a naturally occurring process, however, BMSC migration within a hydrogel is influenced by multiple factors, including mechanical properties such as stiffness, and biochemical cues such as the presence of chemoattractants, or a combination^40^. Signaling molecules play an essential role in guiding pericyte migration toward EC, but the hydrogel stiffness must also permit cellular movement. Migrating pericytes typically express α-SMA^41^. Moreover, HUVEC have been reported to show angiogenic sprouting in the direction of the chemoattractant gradient^42^. It was also reported by Son et al. that fibrinogen concentration, which affects the compressive modulus of the crosslinked matrix, can restrict the angiogenic sprouting and tubular network organization when increased from 2.5 mg/ml. In relation, the fibrinogen used in the current study was 2.0 mg/ml, contributing to the fast sprouting and potential migration in each group^42^.

The developed fibrin-gelatin bioink, vascu-ink, functioned as a 3D bioprinting carrier and 3D environment allowing embedded cells to proliferate, migrate, and produce ECM. Fibrin is a commonly used bioink component in the pursuit of vascularization^9,42^. The fibrin-gelatin bioink exhibited relatively low viscosity, contributing to the high viability of the bioprinted cells. Even after extrusion through a 100 μm nozzle, no qualitative difference was observed between the groups. The variation in nozzle diameter did not affect the alignment or orientation of the HUVEC network, with comparable results across 150 μm, 250 μm, and 500 μm nozzle diameters. Still, the directionality could be influenced by the angiogenic chemoattractant gradient instead, as reported by Son et al.^42^.

Furthermore, the bioink structures enabled the organization of HUVEC into stable vessel-like structures over 14 days in culture, as well as cell migration, and differentiation of BMSC into supportive pericyte cells. The observation that two cell types are able to meet between filaments to associate with and self-assemble the vessel-like structures demonstrates that, in the design of 3D bioprinted or tissue-engineered constructs, EC and PC do not necessarily need to be printed in direct contact, but rather within a migratory distance that allows cellular interaction and structural integration over time. Thus, enabling greater flexibility in bioink and cell localization in advanced biofabrication designs with multiple bioinks and distinct cell types.

H&E and immunohistochemical staining of the explanted constructs and CAM tissue revealed the presence of capillary-like structures within the vascu-ink, providing proof-of-concept that the biomaterial can support endothelial organization toward vascular morphogenesis. CD31 staining demonstrated lumen-like formations surrounded by CD31-positive labeling. Although the CAM assay does not confirm the possibility of perfusion or functional anastomosis, it provides initial evidence that vascu-ink can serve as a permissive microenvironment for early vascular network formation. These findings suggest that vascu-ink may be a promising platform for future vascularized tissue engineering applications.

However, in the future, the presented bioink and co-culture system should be investigated further with respect to functionality and perfusion capabilities^9,43^. Furthermore, in this study, the surface areas of the vasculature were used to compare the vasculogenic capacity across groups, representing a straightforward and reproducible method. In future studies, more delicate methods measuring branch points, junctions, and vessel diameter would be of interest. As well as more advanced measurements of co-localization. Furthermore, any bioink intended for translational use must be evaluated for in vivo functionality, with its components matching the host. Bioprinting facilitates the controlled spatial deposition of cells and biomaterials, enabling the precise design of vessel-supportive microarchitectures and co-culture arrangements. Given the growing potential of multi-material bioprinting and the importance of a functionally vascularized tissue for tissue and transplant survival, the vascu-ink described here could be combined with tissue-specific bioinks to promote vascularization within more complex tissue-engineered constructs.

## Conclusion

The gelatin-fibrin bioink formula supported the spontaneous organization of HUVEC across all groups. However, our findings indicate that direct physical presence of BMSC was critical for robust and stable tubular structure formation, whereas paracrine signaling alone was insufficient to support network formation of comparable size and stability. This underscores the importance of the presence of BMSC and their role as pericytes. Together, these results emphasize the promise of vascu-ink as a suitable matrix for vascular tissue engineering. In future studies, the assessment of vascular functionality, including perfusability, lumen stability, and integration with host vasculature in vivo is needed. Additionally, exploring long-term maturation, perivascular recruitment, and perfusion bioreactor culture may further enhance the translational platform of vascu-ink.

## Supplementary Information

Below is the link to the electronic supplementary material.

Supplementary Material 1

## Data Availability

The datasets generated during and/or analyzed during the current study are available from the corresponding author on request.
