# Supplementary material for: Effect of 3D-printed co-culture design of mesenchymal stem cells and human umbilical vein endothelial cells on tubular formation
Source: Sci Rep. 2026 Jun 8;16:26067. doi: 10.1038/s41598-026-55959-y (PMC13490479; doi:10.1038/s41598-026-55959-y)
Supplement: Supplementary file 1 [file 41598_2026_55959_MOESM1_ESM.docx]

**Supporting information**

**Supplementary materials and methods**

**3D printing with varying nozzle diameters**

The bioink was prepared as reported in section 2.3, and a 5:1 HUVEC:BMSC ratio was used at a cell concentration of 10 million cells/mL. Three different nozzle diameters were used, Ø_ID_ = 0.15 mm (7018443; Nordson EFD, USA), Ø_ID_ = 0.25 mm (7018345; Nordson EFD, USA), and Ø_ID_ = 0.51 mm (7018233; Nordson EFD, USA). The samples were printed as previously described, but a filament distance of 1.4 mm was used instead of 0.75 mm. Samples were printed at pressures of 0.9–2.3 bars and speeds of 8–11 mm/s, depending on nozzle size.

**Supplementary results**

**Figure S1.** Rheology of vascu-ink showing all replicates of hear rate dependency and temperature dependency.

**Figure S2.** a) 3D printed vascu-ink samples printed with different nozzle diameters, and b) immunohistochemical staining for CD31 and nuclei in the 3D printed samples.

**Figure S3.** Metabolic activity measured by CCK-8, represented by the absorbance units at 405 nm. n = 6 for all groups and time points, data presented as mean with error bars representing SD, *p < 0.05, **p < 0.01, *** p < 0.001, **** p < 0.0001 (two‐way ANOVA)

**Figure S4.** Bioprinted samples with only HUVEC and no paracrine effect from BMSC assessed for viability through (a) Live/Dead staining, and for network formation by (b) immunostaining for von Willebrand Factor and Collagen IV
